# Knowledge of, and attitude towards, the treatment of hepatitis C in people who inject drugs

**DOI:** 10.1186/s12954-024-01068-w

**Published:** 2024-08-28

**Authors:** Patrik Roser, Mona Brunstein, Michael Specka, Jörg Timm, Stefan Kühnhold, Fabrizio Schifano, Udo Bonnet, Norbert Scherbaum

**Affiliations:** 1 Department of Psychiatry and Psychotherapy, LVR University Hospital Essen, Medical Faculty, University of Duisburg-Essen, Virchowstr. 174, 45147 Essen, Germany; 2 Center for Addictive Disorders, University Hospital of Psychiatry Zurich, Medical Faculty, University of Zurich, Zurich, Switzerland; 3 Institute of Virology, University Hospital Dusseldorf, Medical Faculty, Heinrich-Heine-University Dusseldorf, Dusseldorf, Germany; 4 Department of Addiction Medicine, LWL Hospital Warstein, Warstein, Germany; 5 Psychopharmacology, Drug Misuse and Novel Psychoactive Substances Research Unit, School of Life and Medical Sciences, University of Hertfordshire, Hatfield, UK; 6 Department of Mental Health, Evangelic Hospital Castrop-Rauxel, Academic Teaching Hospital of the University of Duisburg-Essen, Castrop-Rauxel, Germany

**Keywords:** Hepatitis C, Direct acting antiviral drugs, Attitude, Knowledge, Opioid use disorder

## Abstract

**Background:**

Direct acting antivirals (DAAs) as a curative treatment of hepatitis C have been available for several years and have replaced interferon-containing therapies. However, treatment rates of people who inject drugs (PWID) are declining in Germany, putting the elimination of hepatitis C by 2030 at risk. This study aimed at elucidating the knowledge of, and attitude towards, hepatitis C treatment in a clinical sample of PWID.

**Methods:**

Participants were recruited between February 2019 and October 2020 at two opioid agonist therapy (OAT) clinics and two in-patient drug detoxification wards. Based on the European Addiction Severity Index (Europ-ASI), a standardized interview focusing on: sociodemographic data, drug history, risky behavior, infection with hepatitis C virus (HCV) and HIV, and previous experience with HCV treatment was carried out. In addition, participants filled in a questionnaire evaluating 13 statements relating to HCV treatment (right/wrong) and 15 statements on their personal ‘pros and cons’ views to start such a treatment assessed with the means of a 6-point Likert scale.

**Results:**

A total of 153 patients (average age 45 years, male 78%; 106 (69.3%) currently in opioid maintenance treatment, 47 (30.7%) currently admitted to an inpatient detoxification) with an opioid use disorder were investigated. All of them reported having injected drugs at least once in their lives; 97 participants (63.3%) stated that they had been previously diagnosed with HCV infection. Among them, 27/97 patients (27.8%) reported a previous treatment with interferon; 27/97 (27.8%) with DAAs; and 32/97 (33.0%) reported a currently active hepatitis C. Most patients knew about the availability and efficacy of DAAs. However, DAAs’ low rate of side effects, their short treatment duration, and their replacement of interferon, were not correctly evaluated by up to 50.3% of patients. 25–40% of 32 patients with currently active hepatitis C prioritized handling of social and other medical issues, e.g., reduction of heroin use, over treatment of hepatitis C.

**Conclusions:**

Although current levels of risky behavior have reportedly been reduced by active PWID over the past few years, educational and motivational interventions to increase hepatitis C treatment uptake should address the gaps in patients’ knowledge.

## Background

Hepatitis C is a widespread infectious disease globally affecting about 50 million people, accounting for about 1% of the world population [1]. In Western societies, the major group at risk for hepatitis C virus (HCV) infection is made up by people who inject drugs (PWID). In this group, the hepatitis C prevalence rates are much higher than in other at-risk populations, such as men who have sex with men, with PWID-associated levels amounting to about 55% in North America and 53% in Western Europe [2, 3]. In Germany, an even higher hepatitis C prevalence rate of 67% among PWID was recently reported [4]. Without treatment, about 25% of those affected from an acute infection will spontaneously clear HCV from the body, whereas the remainder progresses to chronic hepatitis C, often ending up in life-threatening diseases such as liver cirrhosis and hepatocellular carcinoma [5]. As no vaccination against hepatitis C is available, treatment of HCV infection is of major value in order to reduce its prevalence. In addition, a widespread of harm reduction interventions such as needle-exchange programs and drug consumption facilities can reduce the risk of infection within the PWID group [6].

Between 2001 and 2011, pegylated interferon (PEG-IFN), in combination with the antiviral drug ribavirin (RBV), was the standard of care of chronic HCV infection [7]. However, this treatment had several limitations, such as a rate of sustained virological response in the range of only 40–50% in genotype 1 and 70–80% in genotypes 2 and 3 [7]. Further limitations included a relatively high frequency of psychiatric, autoimmune and hematological side-effects, and a relatively long treatment period, e.g., up to 48 weeks [8]. Moreover, PEG-IFN is contraindicated in decompensated liver cirrhosis [9] and RBV is contraindicated in renal failure [10]. Due to its limited efficacy and tolerability levels, treatment with PEG-IFN/RBV has been associated with only a rather small effect on reducing the prevalence of hepatitis C [11].

Due to the encouraging scientific progress in the basic understanding of HCV biology, direct-acting antivirals (DAAs) were developed and introduced in 2011; DAAs may present with major advantages compared with the previous PEG-IFN/RBV treatment [12]. In particular, high rates of treatment success exceeding 95%; short duration of treatment (8–12 weeks); a simple administration (e.g., oral tablets); as well as an excellent safety and tolerability profile resulted in the replacement of interferon-based therapies by DAAs [13]. DAAs are equally effective in both PWID and in subjects without any history of injection drug use; hence, these molecules have been recommended for the treatment of chronic HCV infection, regardless of the stage of liver disease [14]. In 2016, based on this paradigm shift in treatment, the World Health Organization (WHO) declared the strategy to reduce the global burden of hepatitis C and to eliminate the disease by 2030 [15]. This strategy includes both the reduction of HCV incidence by 80% and the reduction of HCV mortality by 65%, both to be achieved by increasing HCV screening by at least 90% and HCV treatment by at least 80%.

Despite this remarkable advancement in antiviral therapy, the hepatitis C treatment rate uptake of 52% worldwide and 54% in Europe is still not enough to meet the WHO elimination targets [16]. One could then argue that a high proportion of PWID, and especially those with a basic stabilization of their health and social status due to opioid agonist therapy (OAT), should consider initiating a curative treatment of their hepatitis C with DAAs. In 2021, estimated 81,300 German patients were receiving OAT, accounting for about half of the estimated 160,000 persons dependent on opioids [17, 18].

However, the number of treatment episodes with the new antiviral drugs was so far not sufficient for a sustainable reduction of the prevalence of hepatitis C [4]. After a maximum of more than 20,000 patients (including PWID) treated for their hepatitis C with DAAs in the year 2015 in Germany, the number of treatments dropped consistently over time to about 5600 in 2021 [4]. Taking this development into account, the WHO target to eliminate hepatitis C by 2030 [15] will not be reached, at least in Germany.

It is therefore important to assess the possible obstacles to start treatment, particularly among PWID. In this context, a range of different barriers to screening and treatment of hepatitis C at both a system- and provider-level was identified; these barriers included: lack of decentralized and integrated HCV care services involving specialist and non-specialist HCV treatment providers; insufficient levels of adequate infrastructures; lack of both financial resources and bureaucratic support; and reticence to treat “difficult” patients [19, 20]. At the patient-level, it is speculated whether limited knowledge about HCV, limited awareness of new treatment options, current drug use and/or competing social and mental health issues might represent relevant barriers to HCV treatment [19, 21].

The aim of the study was to assess the knowledge of, and the attitude towards, the treatment of hepatitis C among PWID. Based on a better understanding of potential barriers at the patient-level, a range of proper motivational and psychoeducational interventions could be developed to increase HCV treatment uptake among PWID.

## Methods

### Recruitment

Participants were recruited between February 2019 and February 2020 at two OAT clinics of the University Hospital Essen, Germany, and between January 2020 and October 2020 (with an interruption in April/May due to the Covid-19 pandemic) at two in-patient drug detoxification wards (University Hospital Essen and Psychiatric Hospital Warstein). Study assistants visited facilities on single days. All patients treated at the respective facility on that day were screened for participation. Eligible patients were informed about the study and were asked for participation. Patients aged 18 years or older with a main diagnosis of an opioid use disorder (OUD) according to the international classification of diseases (ICD-10) diagnostic criteria and with at least one episode of intravenous drug use during lifetime were included. Exclusion criteria were: insufficient levels of mastering of the German language, or a current diagnosis of an acute psychosis. Eligible patients were informed about the study, and all patients who agreed to participate to the study provided written informed consent prior to data collection. The study was approved by the Ethics Committee of the Medical Faculty of the University of Duisburg-Essen (Ref. No. 18-8146-BO).

### Instruments

Participants underwent a standardized interview which was based on the German version of the European Addiction Severity Index (Europ-ASI) [22], which was conducted in person by study assistants. The interview asked about sociodemographic information (i.e., age, sex, education, occupational qualification, employment and partnership), information on lifetime and current substance use (i.e., heroin, cocaine, alcohol, benzodiazepines, cannabis and amphetamines), history of OUD treatment (i.e., OAT and/or inpatient detoxification), and psychiatric diagnoses. Moreover, information on risky behavior for HCV infection, such as injection drug use, needle sharing, recurrent use of water, filters or spoons, prostitution and risky sexual behavior, i.e., unprotected sexual activity, was assessed. The interview also included information on the HCV and HIV status of the patients as well as current or previous HCV treatment.

During the interview, participants also filled in a paper questionnaire which evaluated their knowledge about hepatitis C treatment options. From a list of 13 statements relating to hepatitis C treatment, participants were asked to indicate whether a statement was “right” or “wrong”. These statements were particularly related to the spectrum of HCV medications, including DAAs and interferon; the potential of current HCV medication to cure hepatitis C; the side effect profile and duration of HCV therapy; and the possibility to initiate hepatitis C treatment in patients with ongoing alcohol and/or drug use. A second questionnaire consisted of 15 statements referring to the participants’ attitude towards hepatitis C treatment. On the basis of these statements, participants were asked to indicate what reasons might speak for, or against, the treatment of hepatitis C, such as: the individual’s giving priority (or not) to hepatitis C treatment compared to other medical or social issues; the subjectively perceived need for HCV medication; the expectations of medications’ efficacy and side effects; and the possible impact of chronic hepatitis C on health and quality of life. Each of these statements was to be rated on a 6-point Likert scale ranging from “strongly agree” to “strongly disagree”.

Comprehensiveness and practicability of the interview and questionnaires were tested in a pilot study with 12 patients, and questions and the given options of answers were improved where necessary.

### Statistical analyses

Interviews and questionnaires were pseudonymized using a code based on letters derived from patients’ names and their birthdays. The documents were collected at the LVR University Hospital Essen for data entry and statistical analyses. The statistical analyses included descriptive statistics (e.g., percentages, means, and standard deviations) and were performed with IBM SPSS Statistics for Windows, Version 25.0 (Armonk, NY: IBM Corp.).

## Results

### Patients’ characteristics

Out of 312 OUD patients who were treated in the participating facilities on study days, 34 (10.9%) refused participation, 25 (8.0%) were not able to participate due to language problems, 19 (6.1%) due to psychiatric complications, and 21 (6.7%) were missed due to organizational reasons. Another 60 patients (19.2%) indicated no lifetime intravenous drug use. The remaining 153 patients (49.0%) were included into the present study. 106 of the 153 participants (69.3%) were managed in an outpatient OAT and the remaining 47 of the 153 participants (30.7%) were at an inpatient detoxification treatment.

All continuous variables were normally distributed. Participants were on average 45.5 (± 9.1) years-old and mostly male (119/153; 77.8%). The mean duration of OUD was 22.5 (± 10.1) years and the mean duration of the current OAT episode was 53.9 (± 66.7) months. During the previous 30 days, almost every second participant had used heroin (43.8%) and/or alcohol (49.0%), cannabis (32.0%) cocaine (31.4%), benzodiazepines (28.1%), or amphetamines (7.2%). Every other patient reported no current substance use (27/153, 17.0%, of which 26 were in OAT and 1 in naltrexone treatment). About one third of participants (48/153; 31.4%) presented with a comorbid mental disorder, e.g., affective (10.5%), anxiety/stress (9.8%) or personality disorder (13.1%). The sociodemographic and clinical characteristics of the study sample subjects are presented in Table 1.

**Table 1:** Sociodemographic and clinical characteristics of the study sample (N = 153)

*Sociodemographic characteristics*			
Age (in years), mean, SD		45.5	9.1
Sex, n, %	Male	119	77.8
	Female	34	22.2
Education, n, %	Secondary school	102	66.7
	High school	24	15.7
	College	3	2.0
	Primary education	23	15.0
	Unknown	1	0.7
Occupational qualification, n, %	No	69	45.1
	Yes	84	54.9
Employment, n, %	Full-time or part-time	36	23.5
	Odd jobs	24	15.7
	Unemployed	64	41.8
	Other	28	18.4
	Unknown	1	0.7
Partnership, n, %	Yes	42	27.5
	No	81	52.9
	Unknown	30	19.6
*Substance use characteristics*			
Substance use (past 30 days), n, %	None	26	17.0
	Heroin	67	43.8
	Cocaine	48	31.4
	Alcohol	75	49.0
	Benzodiazepines	43	28.1
	Cannabis	49	32.0
	Amphetamines	11	7.2
	Nicotine	143	93.5
	Other	5	3.3
Duration of opioid use disorder (in years), mean, SD		22.5	10.1
Current opioid agonist therapy n, %		106	69.3
Current inpatient detoxification treatment, n, %		47	30.7
Duration of lifetime opioid agonist therapy (in months), mean, SD		119.4	93.9
Duration of current opioid agonist therapy (in months), mean, SD		53.9	66.7
*Psychiatric characteristics*			
Comorbid mental disorder according to ICD-10, n, %	None	103	67.3
	F2 schizophrenia	10	6.5
	F3 affective disorders	16	10.5
	F4 anxiety and stress disorders	15	9.8
	F6 personality disorders	20	13.1
	F9 early onset disorders	3	2.0
	Unknown	2	1.3

F, categories for mental, behavioral and neurodevelopmental disorders of the international classification of diseases (ICD-10)

### Risky behavior for hepatitis C virus infection

Regarding risk behavior for HCV infection, 54 participants (35.3%) reported on injection drug use during the previous 6 months and 49 participants (32.0%) during the last 30 days. The mean duration of injection drug use was 11.4 (± 10.3) years. Needle sharing and recurrent sharing of water, filter or spoons ever in life was reported by 73 (47.7%) and 81 (52.9%) participants, respectively. Prostitution ever in life was reported by 19 participants (13.1%) and risky sexual behavior ever in life by 37 participants (24.2%). The detailed information on risk behavior for HCV infection is given in Table 2.

**Table 2:** Risk behavior for hepatitis C virus infection among the study sample (N = 153)

*Risk behavior for HCV infection*			
Injection drug use, n, %	Yes, lifetime	153	100.0
	Yes, past 6 months	54	35.3
	Yes, past 30 days	49	32.0
Age at first injection drug use (in years), mean, SD		23.3	7.2
Duration of injection drug use (in years), mean, SD		11.4	10.3
Needle sharing, n, %	Yes, lifetime	73	47.7
	Yes, past 6 months	10	6.5
	Never	80	52.3
Recurrent use of water, filters or spoons, n, %	Yes, lifetime	81	52.9
	Yes, past 6 months	10	6.5
	Never	72	47.1
Prostitution, n, %	Yes, lifetime	19	13.1
	Yes, past 30 days	2	1.4
	Never	134	86.9
Risky sexual behavior including no use of condoms, n, %	Yes	37	24.2
	Never	113	73.9
	Unknown	3	2.0

HCV, hepatitis C virus

### Status of hepatitis C virus and HIV infection

Out of the whole study sample, 97/153 participants (63.4%) stated that they had been previously diagnosed with HCV infection. Among them, 27/97 participants (27.8%) reported to have undergone an interferon-based drug therapy, and 27/97 participants (27.8%) had been treated with DAAs. Thirty-four of these 54 subjects (63.0%) reported of a successful HCV treatment. Of those with a history of HCV infection, 30/97 (31%) reported about a spontaneous clearance and 32/97 participants (33.0%) indicated active HCV infection. In addition, 16/153 participants (10.5%) did not know their HCV status and 40/153 participants (26.1%) stated that they had never been infected. With regard to the HIV status, 12/153 participants (7.8%) reported to have tested positive and 2/153 participants (1.3%) declared that they had never been tested. The complete HCV and HIV status of the study sample is given in Table 3.

**Table 3:** Status of hepatitis C virus and HIV infections of the study sample according to self-report (N = 153)

*Hepatitis C*			
HCV status, n, %	Never infected	40	26.1
	Infected	97	63.4
	- Spontaneous clearance	31	20.3
	- Successfully treated	34	22.2
	- Active	32	20.9
	Unknown	16	10.5
HCV treatment, n, %	Interferon-based therapy	27	17.6
	Non-interferon-based therapy	27	17.6
*HIV*			
HIV status, n, %	Negative	136	88.9
	Positive	12	7.8
	Never tested/unknown	5	3.3

HCV, hepatitis C virus; HIV, human immunodeficiency virus

### Knowledge about hepatitis C treatment

**Fig. 1:**
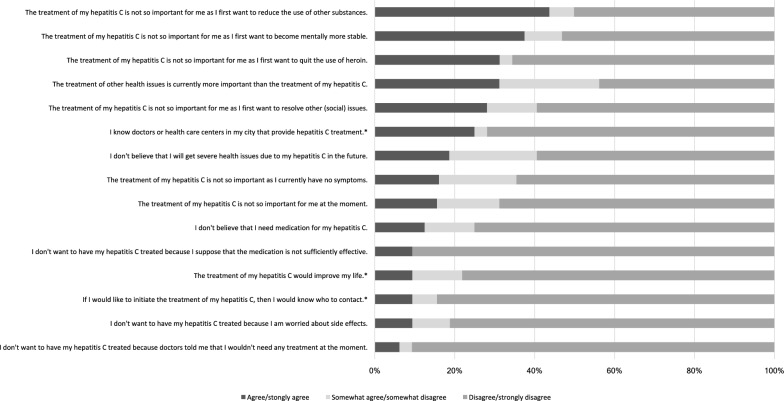
Knowledge about hepatitis C treatment (N = 153), R = right, W = wrong

On average, the study participants (N = 153) answered correctly to 8.6 (± 3.0) out of the 13 statements on hepatitis C treatment. In particular, more than 79.1% gave the correct answer to six statements which covered the following: knowledge about the availability of highly effective drugs for hepatitis C treatment; the potential of HCV medication to treat hepatitis C and prevent long-term complications; and the possibility to treat hepatitis C in patients with a drug use disorder as well. On the other hand, less than 52.9% of the participants were aware of the favorable side effect profile of the currently available HCV medication and the relatively short treatment duration, whereas 51.3% believed that interferon is still the most important drug in the treatment of hepatitis C. Moreover, only less than one third gave the correct answer to the (wrong) statements that hepatitis C treatment “takes about half a year” and that hepatitis C “cannot be treated in patients with alcohol use disorder”. The complete results of the questionnaire are presented in detail in Fig. 1.

### Attitude towards hepatitis C treatment

**Fig. 2:**
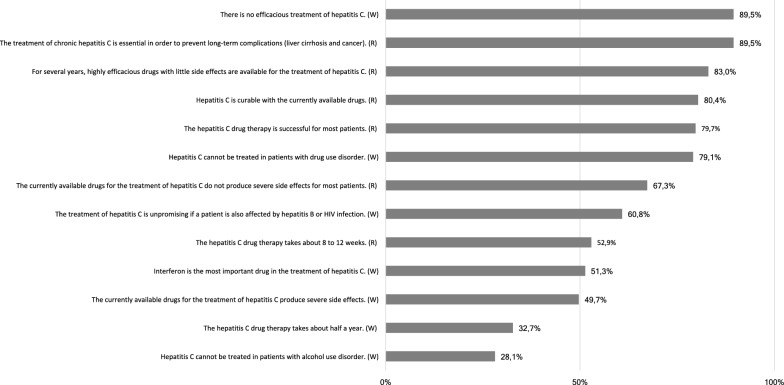
Attitude towards hepatitis C treatment (N = 153), * = reversed-polarity items

32/153 participants reported to currently suffer from active HCV infection. As this subgroup is primarily in need of drug therapy, only the results concerning their attitude towards HCV treatment were analyzed here. The ratings “agree” and “strongly agree” were combined and interpreted as “full agreement”, and the combination of the ratings “disagree” and “strongly disagree” were considered as “full disagreement”. Of note, most participants disagreed with all statements which speak on their attitude against the treatment of hepatitis C, as shown in Fig. 2. This was particularly the case for those statements which related to the medical aspects of the treatment, such as “lack of need for drug therapy”, “insufficient efficacy” and “concerns over side effects”, with each item having been associated with over 70% disagreement. However, a minority of 25–40% of participants agreed with those statements which focused on the priority of medical and/or social issues compared to hepatitis C treatment. Furthermore, they gave a greater importance to the “reduction of heroin use”, “reduction of the use of other substances”, “achievement of mental stability” and “resolving social and other health issues” than to the treatment of hepatitis C. Moreover, 25.0% of the participants replied that they did not know where HCV treatment is provided.

## Discussion

The present study aimed at identifying those factors, at the patient-level, which may contribute to the current gap in hepatitis C treatment uptake in PWID. Therefore, both the knowledge of, and the attitude towards, the treatment of hepatitis C were explored in a sample of PWID diagnosed with OUD. The sociodemographic, substance use and clinical characteristics of the sample were largely comparable with those from a previous study on PWID carried out in Germany [23, 24].

All participants reported a past and/or a current injecting drug use history, which is considered the main mode of transmission of HCV infection in the Western world [1]. Compared to previous data collected between 2011 and 2014 from the same region in Germany [24], the proportion of subjects reporting a drug injection status within the last 30 days decreased here from approximately 86% to 32%. In addition, other important risk factors for transmission of HCV associated with injecting drug use also showed notable reductions over the same period. Specifically, although about 50% of participants had experienced risky behaviors in their lifetime, sharing unsterile needles/syringes and using non-sterile equipment (e.g., water, filters, spoons) in the last 6 months was reported by only 7% of participants, compared with 19% and 36%, respectively, a decade ago [24]. With regard to at-risk sexual behavior, about 24% of the participants reported having engaged in unprotected sexual activity at least once in their lifetime, compared to about 45% in the previous study [24]. Indeed, the proportion of participants who had engaged in prostitution over the past 30 days was rather low, at about 1%. Overall, current results may suggest that injecting and sexual at-risk behavior are still present among PWID, but at much lower levels than previously. Evidence suggests that in the last decade harm reduction interventions, such as the availability of: OAT facilities; needle and syringe exchange programs; drug consumption rooms; free condom distribution; and low-threshold counselling services, have contributed significantly to this positive development [25–27].

Although risky behaviors for HCV transmission within the PWID groups have decreased over the past ten years, there has been only a slight reduction in self-reported HCV prevalence, based on positive test results for anti-HCV antibodies. In this study, 63% of participants disclosed being HCV positive, contrasting with approximately 73% reported between 2011 and 2014 in the same region in Germany [24]. One would however argue that many of current participants were already HCV positive a decade ago, primarily due to their previous high levels of injecting and risky sexual behavior. This may be consistent with the average age of the participants in the current study (46 years) compared to the participants’ average age in the previous study (38 years). Conversely, the marginal decline in HCV prevalence, e.g., of around 10% over the last decade, could be attributed to the observation that new injectors (e.g., those who started these risky practices only in recent years) may have had only relatively small levels of exposure to risky behavior due to the widespread availability of various HCV prevention programs and harm reduction interventions.

The current rate of PWID undergoing hepatitis C treatment (54/97; 56%) is consistent with most recent treatment uptake rates (54%) reported for the European region [16]. This represents a notable increase compared to the treatment uptake rates observed over the past two decades. A systematic review published in 2014 found a median treatment uptake rate of 32% among groups of HCV-RNA positive PWID in the European region [28]. However, it is important to note that the methodology of the current study relied solely on self-reports, without any HCV antibodies/RNA confirmation. Consequently, the treatment uptake rate of 56% in this study encompasses all HCV-positive PWID and is not limited to those who are HCV-RNA positive. Therefore, it is reasonable to infer an even higher treatment uptake rate compared to the findings of the previous systematic review. This positive trend is likely attributed to the widespread and effective implementation of the range of national and international treatment guidelines. These updated guidelines strongly advocate for both expanding low-threshold HCV testing opportunities and providing hepatitis C treatment with DAAs, and especially so for PWID, without any restrictions (e.g., [29]). At this point, it should be noted that, in accordance with the guidelines, all participants in this study who did not know their HCV status or who reported active or unsuccessfully treated HCV infection were referred to HCV screening and treatment, respectively.

In terms of the participants’ understanding of current hepatitis C treatment, the findings revealed considerable variability among the responses provided. On average, approximately two-thirds of the statements regarding hepatitis C treatment were answered correctly, indicating significant levels of knowledge gaps in PWID. For these respondents, particularly challenging were those areas which included certain pharmacological aspects of the antiviral therapy and their contraindications. Nearly half of the participants were unaware that hepatitis C treatment typically lasts only between 8 to 12 weeks, with over two-thirds having assumed a treatment duration of approximately 6 months. This misconception seemed to correlate with the relatively high percentage (around 50%) of participants who felt that interferon remains the primary medication for HCV treatment. This finding may suggest a notable gap in knowledge regarding the shift from interferon-based treatments to DAAs [12]. Present concerns regarding interferon treatment and limited awareness of newer HCV treatment options among PWID were echoed in several recent qualitative studies from the United Kingdom and the United States [30–33]. Another unexpected finding from this study was the misconception held by more than 70% of participants that hepatitis C cannot be treated in individuals with alcohol use disorder. It remains speculative whether these participants based their assumption on the hepatotoxic effects of alcohol, which could potentially counteract the therapeutic benefits of antiviral therapy.

Regarding attitudes towards hepatitis C treatment, only responses from participants who reported having an active HCV infection were analyzed. Most respondents from this subgroup disagreed with those statements opposing hepatitis C treatment. However, as many as 40% of them expressed concerns about remaining factors such as drug use, mental health issues, and social problems, suggesting that these issues should be addressed before initiating hepatitis C treatment. This perspective appears to be prevalent among HCV-positive PWID, as previously observed in attitudes towards HCV screening [32–34], once again indicating a potential gap in knowledge regarding eligibility criteria for HCV treatment. In fact, current drug use, comorbid mental disorders, and social challenges should not automatically disqualify individuals from receiving HCV treatment. Instead, treatment should be accompanied by multidisciplinary support tailored to individual needs, enabling simultaneous addressing of various areas of concern [29].

Several limitations need to be considered when interpreting current findings. Firstly, the research was conducted exclusively in two addiction medicine facilities located in the federal state of North Rhine-Westphalia in Western Germany. Consequently, the findings may not be generalizable to populations of PWID from either remaining German regions or different countries, where different preventive measures and educational programs could have been implemented. Secondly, data regarding patient characteristics, such as substance use, risky behavior, and HCV status, relied on self-reported information only, without any further laboratory-based verification. However, previous addiction medicine studies have consistently shown that operational self-reports are generally valid, and particularly those concerning both levels of illicit drug use and HCV infection status [35, 36]. Additionally, the primary aim of this study was to investigate barriers to hepatitis C treatment among PWID rather than to collect detailed data relating to their HCV status. Thirdly, there may have been significant selection biases associated with the recruitment of study participants. It is possible that PWID with more severe addiction issues and functional impairments either declined participation in the study or provided unreliable information due to lack of motivation or understanding of the questions.

## Conclusions

In summary, this study has highlighted several barriers to HCV treatment at the patient-level; these included limited levels of knowledge relating to availability of HCV medications and the low-threshold eligibility criteria for this treatment provision. These findings underscore the urgent need to enhance efforts in motivating and educating PWID, which is crucial for bridging the gap in hepatitis C treatment uptake and achieving the WHO elimination targets. Psychoeducational interventions should focus particularly on the current HCV treatment options utilizing DAAs. A range of appropriate psychoeducation initiatives should be delivered not only through specialist centers but also within both the OAT facilities and the community-based outreach programs; these initiatives have demonstrated efficacy in boosting both HCV testing and treatment uptake, as well as enhancing treatment retention levels whilst achieving sustained virological response during antiviral therapy [37–39]. Furthermore, we suggest that integrating peer support within a multidisciplinary care framework can prove to be an effective strategy in addressing knowledge gaps, hence addressing concerns about HCV treatment, and ultimately increasing treatment acceptance levels [40, 41]. And finally, the results of this study indicate that, alongside harm reduction measures and multidisciplinary care, close interdisciplinary collaboration among medical specialists for addiction medicine, psychiatry and hepatology including direct referral of patients to HCV screening and treatment is crucial in order to overcome barriers in HCV elimination for PWID.

## Data Availability

Study datasets are available from the corresponding author on reasonable request.

## Abbreviations

ASI: Addiction severity index
ICD: International classification of diseases
DAA: Direct acting antiviral
PWID: People who inject drugs
HCV: Hepatitis C virus
HIV: Human immunodeficiency virus
PEG-IFN: Pegylated interferon
RBV: Ribavirin
WHO: World Health Organization
OAT: Opioid agonist therapy
OUD: Opioid use disorder
